# Bilateral Apocrine Hidrocystomas in the Lower Conjunctival Fornix: A Case Report

**DOI:** 10.7759/cureus.90343

**Published:** 2025-08-17

**Authors:** Kosuke Minami, Hideki Fukuoka, Chie Sotozono

**Affiliations:** 1 Department of Ophthalmology, Kyoto Prefectural University of Medicine, Kyoto, JPN

**Keywords:** apocrine, apocrine hidrocystoma of the conjunctiva, apocrine secretion, conjunctival cyst, pathological diagnosis

## Abstract

We report the case of an elderly patient who presented with an apocrine hidrocystoma in the lower conjunctival fornix in both eyes. A 75-year-old woman exhibited tumors with a serous component, partly blue in color, in the bilateral lower conjunctival fornix in both eyes. A biopsy of the tumor in the conjunctival fornix in the left eye revealed the formation of multilocular cysts in the subepithelial stroma, lined with several layers of cuboidal epithelium, and papillary growth into the cyst lumen was also evident. Thus, a definitive diagnosis of an apocrine hidrocystoma was made. The findings in this case highlight that apocrine hidrocystomas can occur bilaterally in the lower conjunctival fornix.

## Introduction

Cystic disorders that commonly occur in the conjunctiva include epidermoid cysts, epithelial inclusion cysts, and various conjunctival cysts (i.e., lymphoid, inclusion, and retention cysts). Similarly, skin-related cystic abnormalities of the eyelid encompass conditions such as sweat gland cysts and sebaceous gland cysts, which originate from skin appendages [1]. However, it is uncommon for those cysts to occur in the conjunctival tissue beyond the tarsal plate or extraocular muscles. Specifically, apocrine glands, a type of sweat gland, are typically abundant in the axilla and can also be found in regions such as the external ear canal and nasal alae, with openings usually at the upper portion of hair follicles.

In the field of ophthalmology, the gland of Moll, a ciliary gland located in the eyelid margin, is recognized as an apocrine sweat gland. First reported by Mehregan [2] in 1964, apocrine sweat gland cysts, i.e., benign cystic tumors that originate from apocrine sweat glands, are believed to occur in regions such as the uvula, ear, chest, and shoulder [3,4], and although they can develop in the periorbital area and eyelids, it is exceedingly rare for them to occur in the conjunctiva.

In this report, we present the case of an elderly patient who was diagnosed with an apocrine hidrocystoma in the lower conjunctival fornix in both eyes.

## Case presentation

This study presented a 75-year-old woman who initially sought medical consultation at a nearby ophthalmology clinic due to bilateral foreign body sensation and excessive tearing. Upon examination, she was diagnosed with allergic conjunctivitis and was prescribed oral antibiotics and low-dose steroid eye drops for treatment. However, the symptoms did not improve, and because no clinical improvement was observed and neoplastic lesions such as lymphoma were also suspected, she was ultimately referred to the Department of Ophthalmology at the Kyoto Prefectural University of Medicine Hospital, Kyoto, Japan.

**Figure 1:**
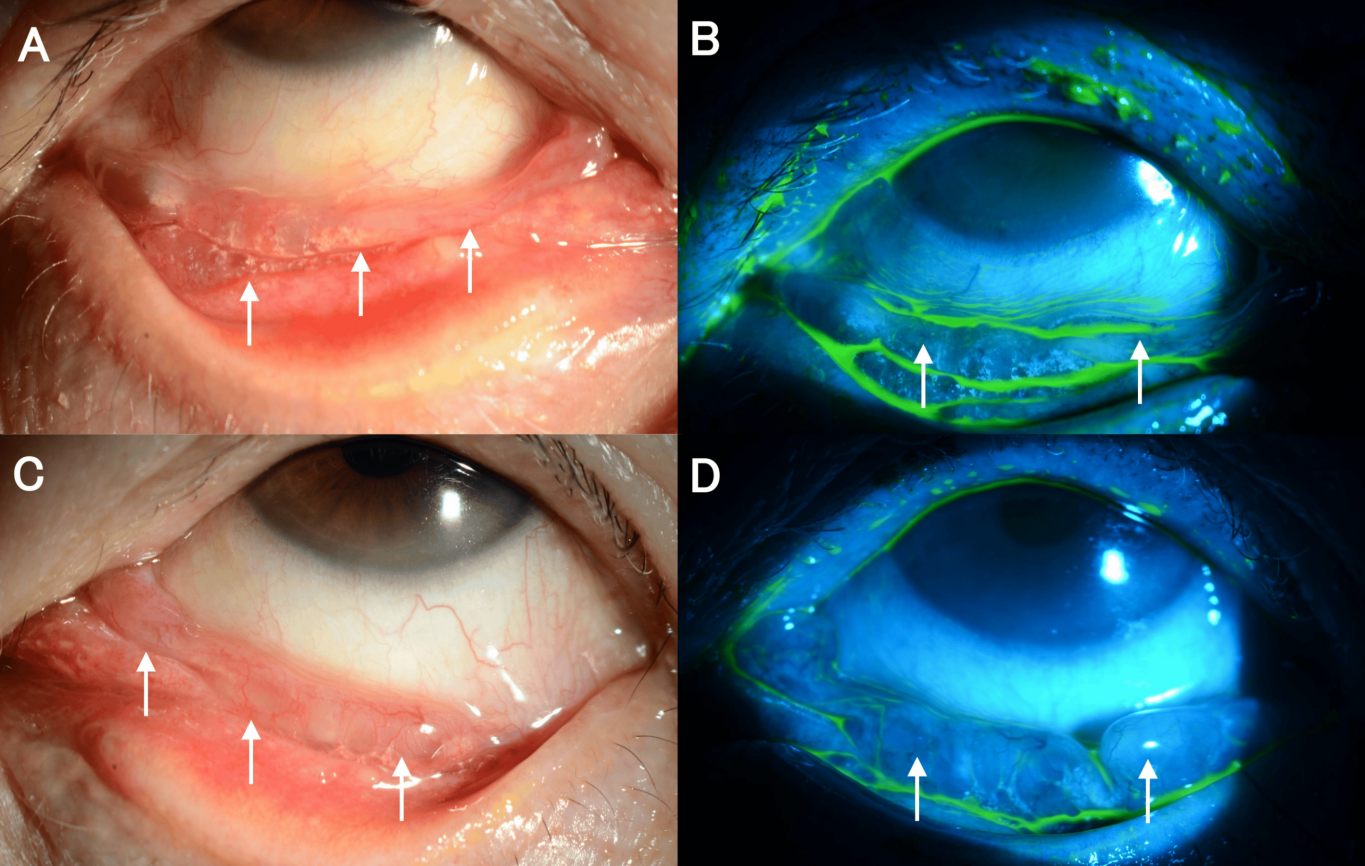
Slit-lamp photographs of the patient's eyes at initial presentation. (A) Elevated lesion suggestive of serous fluid-filled tumor was observed in the lower conjunctival fornix of the right eye (white arrows). (B) Slit-lamp photograph of the right eye with fluorescein showing the lesion (white arrows). (C) An elevated lesion suggestive of a serous fluid-filled tumor was observed in the lower conjunctival fornix of the left eye (white arrows). (D) Slit-lamp photograph of the left eye with fluorescein showing the lesion (white arrows).

A thorough examination revealed best-corrected visual acuity of 1.0 (20/20) in both eyes. Intraocular pressure was 13.7 mmHg in the right eye and 16.0 mmHg in the left eye. The patient had a history of cataract surgery in both eyes. We observed what appeared to be tumors containing serous fluid in the lower conjunctival fornix of both eyes (Figure 1). Fundus examination showed no abnormal findings.

**Figure 2:**
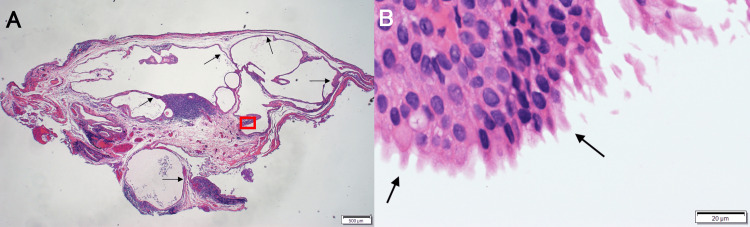
Histopathological image of elevated lesions in the lower conjunctival fornix, suspected to be serous fluid-filled tumors. (A) Image showing the development of cystic formations with multiple chambers in the subepithelial stroma of the conjunctiva, with the inner surfaces (black arrows) of the cysts covered by several layers of cuboidal epithelium. Papillary growth into the cystic cavity can also be seen, and secretion was observed upon sectioning (hematoxylin and eosin stain: ×12.5 magnification). The red box indicates the area shown at higher magnification in panel B. (B) Higher magnification view of the boxed area in panel A showing decapitation secretory cells indicative of apocrine secretion (black arrows) observed in the cyst lumen (hematoxylin and eosin stain: ×400 magnification).

On the same day, a biopsy of the tumor in the lower conjunctival fornix in the left eye was performed, as neoplastic lesions such as lymphoma were also suspected. A histological examination of the tissue specimen revealed the formation of multilocular cysts in the subepithelial stroma, lined with several layers of cuboidal epithelium. Papillary growth into the cyst lumen was also evident, and a diagnosis of apocrine hidrocystoma was made (Figure 2).

No malignant features were identified, and since the patient did not wish to undergo complete excision of the adenoma in both eyes, she has subsequently been undergoing scheduled follow-up examinations. The patient has been followed for 10 years, with no evidence of adenoma recurrence or further enlargement.

## Discussion

Apocrine hidrocystoma is a benign cystic tumor first reported and established as an independent disease by Mehregan [2] in 1964 after the collection of 17 cases, and it likely arises in the uvula, ears, chest, shoulders, periocular regions such as the eyelid, and sclera [5,6].

To date, there have been only two reported conjunctival hidrocystoma cases in Japan: one in which it occurred in the tarsal conjunctiva of the eyelid [7] and another in which it occurred in the bulbar conjunctiva [8]. Moreover, there have been five reported cases internationally: two that occurred in the inner corner of the eye [9] and three that occurred in the bulbar conjunctiva [10-12]. However, it should be noted that in those seven reported cases, the development of the cysts was unilateral, not bilateral. After conducting a literature review by utilizing PubMed and Google Scholar and using the key words multiple/bilateral conjunctival apocrine hidrocystoma, we did not find any reports of bilateral conjunctival apocrine hidrocystoma.

Apocrine hidrocystomas arise from the tumorous proliferation of apocrine sweat glands and are most commonly located in close proximity to hair follicles in regions such as the scalp, axillae, external auditory canal, eyelids, nasal alae, areola and nipple, perineum, and external genitalia. Given that apocrine glands are most densely distributed on the scalp and face, these sites represent the most frequent locations for apocrine hidrocystomas [13]. However, it is believed that the higher prevalence of apocrine hidrocystomas around the eyes is due to their origin from the gland of Moll, typical for the periorbital region, and in this present case, the hidrocystomas were found in the conjunctiva of both eyes, where the glands of Moll are not usually found. However, rare reports of hidrocystoma occurring in areas where apocrine glands are not typically present, such as the forearm, suggest the possibility that apocrine hidrocystomas can develop in various locations throughout the body [14].

Typical histological findings include an inner layer composed of cuboidal to columnar cells with acidophilic cytoplasm at the tips and a myoepithelial cell layer. Moreover, apocrine sweat glands often exhibit the phenomenon of decapitation secretion, characterized by the basal constriction of cell protrusions that project into the lumen, causing droplet-like fragments of cytoplasm to be released.

In this present case, papillary growth into the cyst lumen and evidence of decapitation secretion led to our diagnosis of apocrine hidrocystoma. These hidrocystomas can exhibit a variety of colors, ranging from flesh-colored to gray and even bluish-black. In cases with a bluish-gray coloration, it is attributed to a high lipid content, thus causing the Tyndall effect [5]. In the case presented in this study, a portion of the hidrocystoma appeared bluish, consistent with the characteristic coloration of lipofuscin.

It should be noted that differential diagnoses can include eccrine hidrocystoma, conjunctival inclusion cysts, epidermoid cysts, and other similar conditions, but these can usually be distinguished based on histopathological findings. As stated above, apocrine hidrocystomas are benign, and malignant transformation is exceptionally rare. In chronic inflammatory conjunctival diseases, conjunctival cysts are commonly observed. However, in this case, both eyes exhibited tumorlike lesions containing serous fluid spanning the entire lower conjunctival fornix. Unlike the clear fluid typically seen in conjunctival cysts, the lesions contained slightly bluish fluid. Given these features, the lesions were considered atypical for conjunctival cysts, and a biopsy was performed rather than simple observation.

The usual treatment is surgical excision. Charles et al. treated the cyst by puncturing it and draining the fluid, but the fluid reaccumulated within three months [10]. In this case, the partial excision of the cyst in the left eye was performed, whereas the complete excision of both eye lesions was declined by the patient and managed with observation. No recurrence of the left eye cyst has been noted during 10 years of follow-up.

## Conclusions

In conclusion, while previous reports have documented cases of apocrine hidrocystomas occurring unilaterally, the patient in this report represents the first known case in which apocrine hidrocystomas developed bilaterally in the lower conjunctival fornix. This presentation offers a novel perspective on the potential distribution and occurrence patterns of apocrine hidrocystomas in conjunctival tissue. The bilateral nature of this condition suggests the potential contribution of systemic factors or developmental anomalies to hidrocystoma formation, necessitating further investigation into the underlying mechanisms. Although apocrine hidrocystomas are benign lesions with an excellent prognosis, their occurrence in atypical locations, such as the conjunctiva, necessitates meticulous histopathological examination for precise diagnosis and suitable management. This case underscores the significance of incorporating apocrine hidrocystoma into the differential diagnosis of bilateral conjunctival cystic lesions, particularly when characteristic histological features, such as decapitation secretion and papillary growth, are evident.
